# Platelet-rich plasma (PRP) accelerates murine patellar tendon healing through enhancement of angiogenesis and collagen synthesis

**DOI:** 10.1186/s40634-020-00267-1

**Published:** 2020-07-08

**Authors:** Yohei Kobayashi, Yoshitomo Saita, Tomoiku Takaku, Tomomasa Yokomizo, Hirofumi Nishio, Hiroshi Ikeda, Yuji Takazawa, Masashi Nagao, Kazuo Kaneko, Norio Komatsu

**Affiliations:** 1 Department of Orthopaedics, Juntendo University Faculty of Medicine, Tokyo, Japan; 2 Department of Hematology, Juntendo University Faculty of Medicine, 2-1-1 Hongo, Bunkyo-ku, Tokyo, 113-8421 Japan; 3 International Research Center for Medical Sciences, Kumamoto University, Kumamoto, Japan

**Keywords:** Platelet-rich plasma (PRP), Tissue healing, Patellar tendon

## Abstract

**Purpose:**

Although platelet-rich plasma (PRP) therapy has become an increasingly popular treatment for sports-related injuries, the molecular mechanisms of PRP on tissue healing process remain poorly understood. The aim of the present study was to develop an experimental method quantifying the efficacy of PRP with murine patellar tendon injury model, leading to future elucidation of the mechanisms of PRP on healing processes.

**Methods:**

Full-thickness defects were created in the central third of the murine patellar tendon. The prepared allogenic PRP gel was applied on the defect of the patellar tendon (PRP group), while the remaining mice served as the untreated control group. Mice were sacrificed at 2, 4, 6, 8, and 10 weeks after the operation, with histological sections obtained in each time point (*n* = 4 / time point / group). Semi-quantitative histological evaluation was performed in accordance with the Bonar score. The variables included in this scoring system were cell morphology, ground substance, collagen arrangement, and vascularity, with higher grades indicating worse tendon structures. In addition, the ratio of the collagen fibers to the entire tendon tissue (FT ratio) was measured using KS400 software as a quantitative histological evaluation.

**Results:**

The total Bonar score in the PRP group was significantly lower than in control group. With regard to the variables in the Bonar score, the vascularity score was significantly higher in the PRP group at 2 and 4 weeks, while the collagen arrangement score was significantly lower in the PRP group at 8 weeks. Based on a quantitative evaluation, the recovery speed of the patellar tendon determined by FT ratio was significantly faster in the PRP group than in the control group at 6 and 8 weeks.

**Conclusions:**

We have developed an experimental method for histological and quantitative evaluation of the effects of PRP on tissue healing using murine patellar tendon injury model. The results of this study suggest that the local application of PRP could enhance the tissue-healing process both directly through action on localized cells and indirectly through the recruitment of reparative cells through the blood flow. Further investigations will be needed to confirm the mechanisms of PRP in tissue-healing processes with the development of this experimental model.

## Background

Platelet-rich plasma (PRP) is an autologous platelet concentrate that contains diverse growth factors, such as platelet-derived growth factor (PDGF), transforming growth factor-beta (TGF-β), vascular endothelial growth factor (VEGF), epidermal growth factor (EGF), fibroblast growth factor (FGF), and insulin-like growth factor 1 (IGF-1) [1, 2]. PRP therapy hold much promise as a simple, safe (because of its autologous origin), low-cost, and minimally invasive technique that would promote tissue healing processes [2–4]. PRP therapy was initially introduced in maxillofacial and plastic surgery in the 1990s [5] and in many other fields subsequently [6–8]. Recently, PRP therapy has been used as one of the therapeutic applications for orthopaedics and sports-related injuries [2, 9–11].

Despite an increasing number of both clinical and basic studies that support the efficacy of PRP therapy [12–16], some studies have shown less favorable results [17, 18]. Moreover, there exist several problems related to the use of PRP therapy, and the evidence supporting the use of PRP in the clinical setting remains insufficient. One of the problems encountered in the clinical use of PRP is that its molecular mechanisms of PRP in the tissue-healing process remain poorly understood. In a previous study, Lyras et al. reported that the administration of PRP significantly increased angiogenesis during the early phase of the tendon-repair process using a rabbit Achilles tendon injury model [16]. However, the cells participating in the early phase of the PRP-dependent tissue-repair process have not been identified in this report. An elucidation of the mechanisms of the tissue-healing process promoted by PRP is essential to establish evidence for PRP therapy. Hast et al. reviewed the role of animal models in tendon research [19]. In this review, they described that animal models could provide the ability to reproduce consistent and repeatable injuries that can be treated in a controlled and quantifiable manner, as well as allow the evaluation of invasive treatments and assessments that would be unethical in human subjects. Moreover animal models have the capability of modifying the genome particularly in the murine model. This technology allows the comparison of tissue properties in mice with and without the ability to express a particular gene globally. To the best of our knowledge, none of the previous in vivo studies regarding the efficacy of PRP have used a murine patellar tendon injury model. Therefore, the aim of this study was to develop an experimental method quantifying the efficacy of PRP with a murine patellar tendon injury model, leading to the future elucidation of the mechanisms of PRP in healing processes.

## Methods

### Experimental design

A total of 51 female C57BL/6 J mice (12-week old) purchased from The Charles River Laboratories Japan (Kanagawa, Japan) were used in this study. The effects of PRP application on the created defect of the murine patellar tendon was investigated histologically in comparison with the untreated control group at five different time points (2, 4, 6, 8, and 10 weeks) after the surgery (*n* = 4 / time point / group). One mouse was used to obtain the unoperated control specimens for the parallel histologic evaluations. The remaining 10 mice were used as donor of PRP.

### PRP preparation

PRP was obtained from the whole blood of female C57BL/6 J donor mice (12-week old, *n* = 10) through the double-spinning method. Briefly, approximately 1 ml of whole blood was drawn via cardiac puncture into a microtube (Microtainer, BD Biosciences, Bedford, MA, USA) containing EDTA-2 K as an anticoagulant. After the first centrifugation step of 400 g for 10 min at 25 °C, the upper layer and buffy coat were transferred to another tube. After the second centrifugation step of 2000 g for 3 min at 25 °C, the supernatant (platelet-poor plasma, PPP) was collected. The settled pellet containing platelets and leukocytes was resuspended in the remaining 100 μl of plasma volume to produce PRP. This buffy coat-based PRP preparation method has been previously reported by some authors [20, 21].

### Hematological analysis

The platelet, leukocyte, and erythrocyte concentrations and leukocyte compositions from the whole blood and from each of the PRP and PPP samples were determined using an automated hematology analyzer (Poch-100iV Diff, Sysmex, Kobe, Japan) immediately after preparation. After this analysis, all samples were stored at − 80 °C until further analysis and application.

### Quantification of the growth factors

The concentrations of the three representative growth factors for tissue repair (PDGF-AB, VEGF, and TGF-β1) from some of the stored PRP and PPP samples (4 PRP and 4 PPP from the same donor) were measured using double antibody sandwich enzyme-linked immunosorbent assay kits (R&D Systems, Minneapolis, MN, USA) according to the manufacturer’s instructions. Before quantification, a single freeze–thaw cycle was used to induce platelet activation and release of growth factors. The samples were thawed and then incubated for 1 h at 37 °C. After incubation, the samples were centrifuged for 5 min at 13,000 g and the supernatants were tested. For the TGF-β1 assay, the samples were first treated with an acid solution (1 N HCl) to activate the latent TGF-β1 to the immunoreactive form. Prior to the assay, the samples were diluted with buffer to avoid the matrix effects. All PPP samples were diluted twofold regardless of the type of growth factor, whereas the PRP samples were diluted in each dilution depending on the type of growth factor (PDGF-AB, VEGF: 50-fold, TGF-β1: 100-fold, respectively). All samples were measured in triplicate and the OD values were measured under 450 nm wavelength absorbance using a microplate reader (Bio- Rad, Hercules, CA, USA). The sample concentrations were obtained by interpolating from the standard curve.

### Surgical procedure and PRP application

Full-thickness defects were created in the central third of the patellar tendon of the right hindlimbs via microsurgery technique as described by Dyment et al. [22, 23]. Briefly, after anesthetization with 4% isoflurane, a longitudinal skin incision was made on the right hindlimb over the patellar tendon. A microtweezer was then slid under the tendon and spread to exert tension on the tendon. The lateral and medial edge of the defect was created using a scalpel, and then the central strip of the tendon was excised at the proximal and distal insertions with microscissors. The stored frozen allogenic PRP was thawed and 0.5 mM calcium chloride was added (Sigma-Aldrich, St. Louis, MO, USA), after which it was incubated for 1 h at 37 °C to activate and cause gelling of the PRP. The prepared allogenic PRP gel (50 μl) was applied on the defect of the patellar tendon in 20 mice (PRP group), while the remaining 20 mice served as the untreated control group (control group). At the end of the procedure, the skin was closed with a 5–0 nylon suture. The mice were allowed full activity in their individual cages following surgery until euthanasia for histological analysis.

### Histological analysis

The mice were sacrificed at 2, 4, 6, 8, and 10 weeks after the operation and frozen histological sections were obtained in each time point (*n* = 4/time point/group) according to the method described by Kawamoto et al. [24]. After euthanasia, the femur and tibia were cut midshaft and the skin removed. The samples were cryoprotected with 30% sucrose (ATAGO, Tokyo, Japan) overnight at 4 °C after fixation with 4% paraformaldehyde (Nakarai Tesque, Kyoto, Japan) for 1 h at 4 °C. After washing three times with 1x PBS, the samples were frozen in cooled hexane (Nakarai Tesque, Kyoto, Japan), and then freeze embedded with carboxymethyl cellulose (CMC) gel (Leica Microsystems, Wetzlar, Germany) in the coolant. The adhesive film was applied on the cutting surface of the CMC block, and then sagittal frozen sections (5 μm thickness) were made within the defect with a tungsten carbide blade. The normal tendon specimens obtained from the unoperated female C57/B6J mouse (12-week old, *n* = 1) were sectioned in comparable locations. The serial frozen sections from each sample were stained with hematoxylin and eosin (H&E), Alcian blue, and Azan to visualize the tissue morphology. Images were taken using a digital camera attached to a light microscope (AX73, Olympus, Tokyo, Japan). Semi-quantitative histological evaluation of the tendon-healing process was performed in accordance with the Bonar score [25, 26]. The variables included in this scoring system were cell morphology, ground substance, collagen arrangement, and vascularity, with higher grades indicating worse tendon structures (each factor; 0–3 points, total score; 0–12 points). One pathologist blindly scored one representative section from each sample stained with H&E, Azan, and Alcian blue for those parameters. In addition, the ratio of the collagen fibers stained with Azan to the entire tendon tissue (defined as “FT ratio”) was measured using the KS400 image analyzing software (Carl Zeiss, Oberkochen, Germany) for quantitative histological evaluation, as previously described by Sugihara et al., Kajimoto et al., and Schipke et al. [27–29]. Briefly, the line manually drawn along the tendon periphery on one representative Azan section from each sample by one operator (YK) using the software was set as the region of interest (ROI). Then, percentages of the collagen fiber area positively stained with Azan in the ROI were determined by computer-assisted morphometry, with a high percentage indicating a better tendon structure containing rich collagen fiber as closer to a normal tendon.

### Statistical analysis

All data were presented as mean ± SD. Comparisons of all pairs in each group were conducted using unpaired Student’s t test after the distribution of normality using the Shapiro–Wilk test. Mean values were compared by one-way analysis of variance followed by a post hoc Tukey–Kramer multiple comparison test for more than three groups. All *p*-values were two-sided and p-values less than 0.05 were considered to be statistically significant. Statistical analyses were performed using the GraphPad Prism version 6.0 software package (GraphPad Software, Inc., La Jolla, CA, USA).

## Results

### Establishment of a murine patellar tendon injury model

**Fig. 1:**
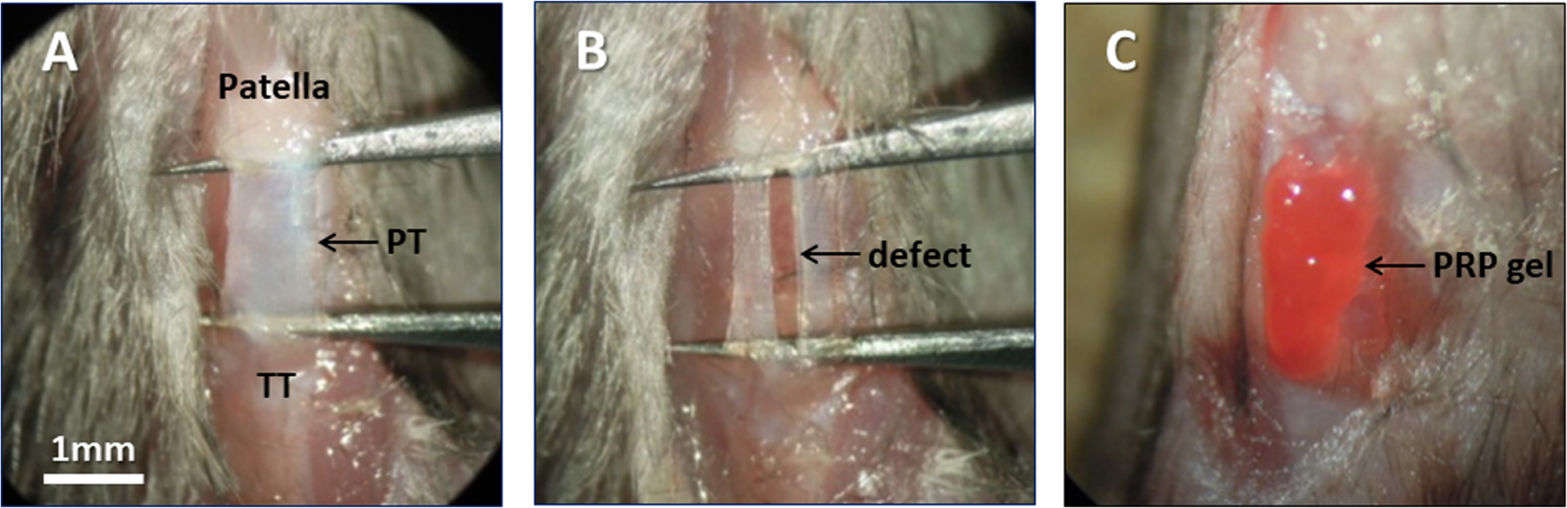
Surgical procedure and PRP application. (**a**, **b**) Full-thickness defect was created in the central third of the patellar tendon. **c** Platelet-rich plasma (PRP) gel was applied on the defect of the patellar tendon. PT: patellar tendon. TT: tibial tuberosity

We performed surgeries to create a patellar tendon injury, known as the ‘window defect’ model. Full-thickness defects were created in the central third of the murine patellar tendon (Fig. 1a, b). The surgeries recorded a 100% success rate, without any lameness or tendon rupture following the operations. The tendon defect width (0.32 ± 0.07 mm) averaged 29.1% of the total patellar tendon width (1.10 ± 0.13 mm). There were no significant differences in the defect width and defect ratio (defect width/total width) between the PRP group and the control group (*p* = 0.62 and *p* = 0.73, respectively). After the surgery, the prepared allogenic PRP gel was applied on the defect in the PRP group (Fig. 1c).

### PRP characterization

We performed the hematological analysis and the quantification of growth factors as the PRP characterization.

**Fig. 2:**
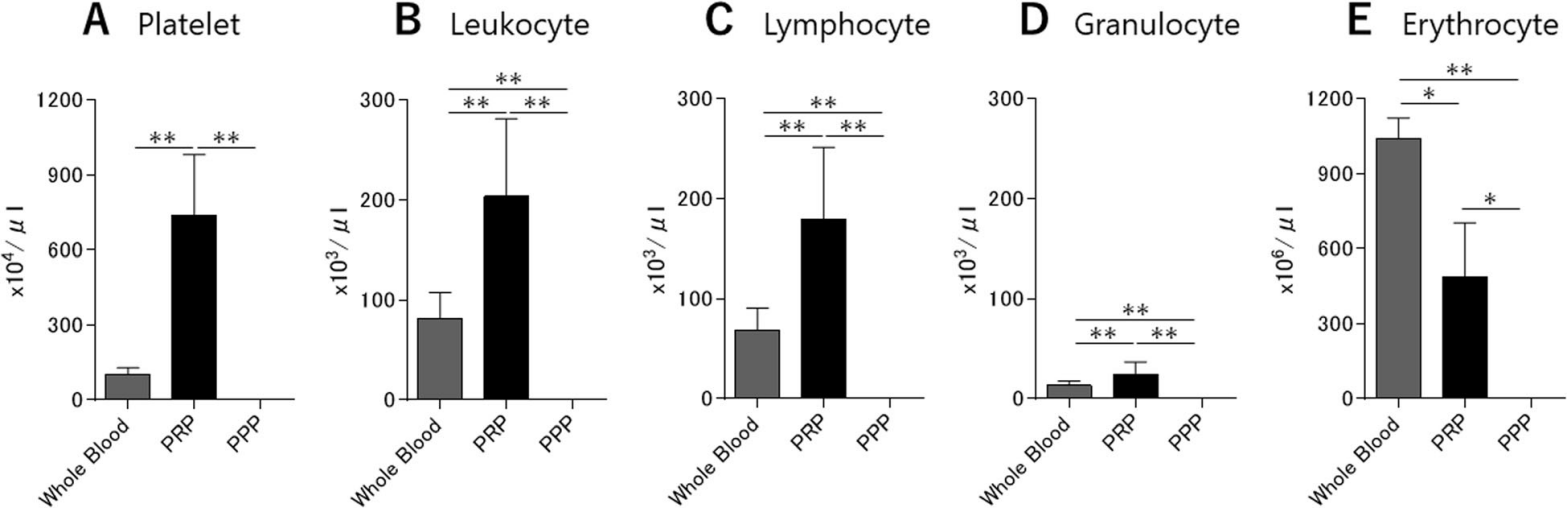
Hematological analysis. **a** Platelet concentrations, **b** leukocyte concentrations, **c** lymphocyte concentrations, **d** granulocyte concentrations, and (**e**) erythrocyte concentrations of whole blood, PRP, and platelet-poor plasma (PPP) (*n* = 10). Data are presented as mean ± SD (*p < 0.05, **p < 0.01)

The platelet concentration of the PRP was approximately 7.1-fold higher than that of the whole blood, whereas PPP contained very few platelets. Three were significant differences between the whole blood and the PRP (*p* < 0.001) and between the PRP and the PPP (*p* < 0.001) (Fig. 2a). The leukocyte concentration of the PRP was approximately 2.5-fold higher than that of the whole blood, whereas the PPP contained very few leukocytes. Three were significant differences between the whole blood and the PRP (*p* < 0.001), between the PRP and the PPP (*p* < 0.001), and between the whole blood and the PPP (*p* = 0.002) (Fig. 2b). In both the whole blood and the PRP, the composition of the leukocyte was predominantly lymphocyte (83.6% and 88.2%, respectively), followed by granulocyte (Fig. 2c, d). The erythrocyte concentration of the PRP was approximately 0.5-fold lower than that of the whole blood, whereas the PPP contained very few erythrocytes. Three were significant differences between the whole blood and the PRP (*p* < 0.001), between the PRP and the PPP (*p* < 0.001), and between the whole blood and the PPP (*p* < 0.001) (Fig. 2e).

**Fig. 3:**
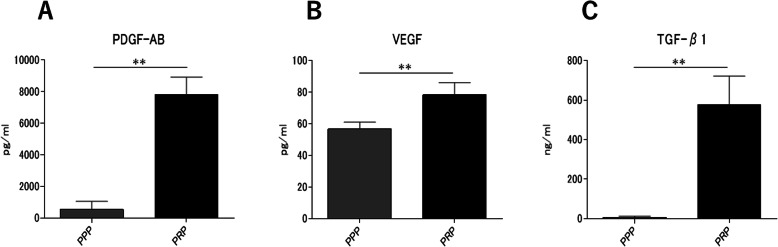
Growth factor concentrations in PRP and PPP. **a** Platelet-derived growth factor-AB (PDGF-AB), **b** vascular endothelial growth factor (VEGF), and (**c**) transforming growth factor-beta 1 (TGF-β1) concentrations of PRP and PPP (*n* = 4). Data are presented as mean ± SD (**p < 0.01)

The concentrations of PDGF-AB, VEGF, and TGF-β1 were all significantly higher in the PRP than in the PPP (*p* < 0.001, *p* = 0.005, *p* < 0.001, respectively). However, the magnification differed depending on the type of growth factor (approximately 14.6-fold, 1.4-fold, and 85.6-fold, respectively) (Fig. 3a-c).

### Time-course analysis of PRP-treated injury

To analyze and evaluate the recovery from injury, we performed histological analysis at different time points.

**Fig. 4:**
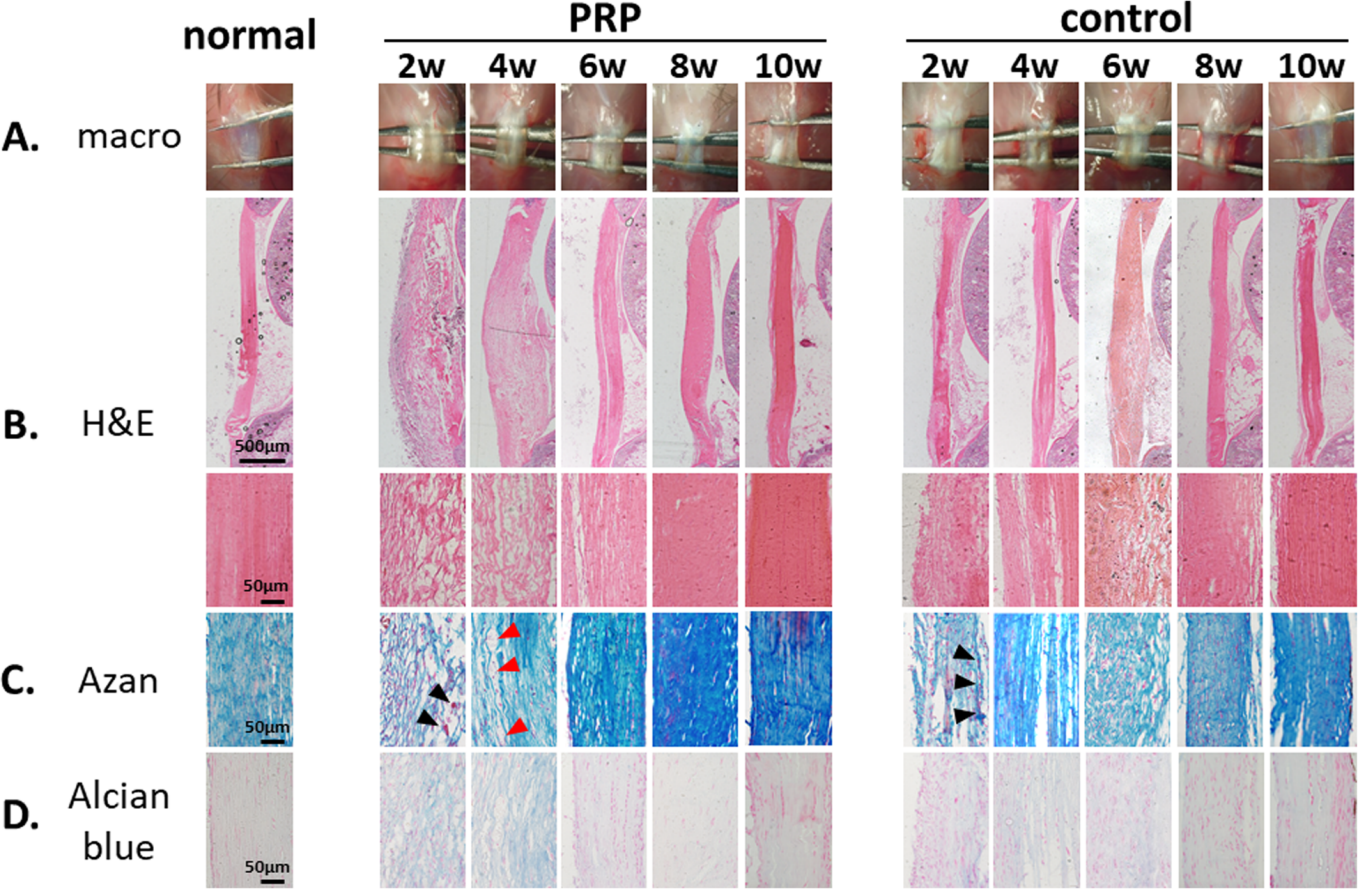
Representative macroscopic and histological findings. Representative (**a**) macroscopic findings and serial sagittal sections stained with (**b**) hematoxylin and eosin (H&E), (**c**) Azan, and (**d**) Alcian blue of normal patellar tendon (left column), PRP group (middle column; 2, 4, 6, 8, and 10 weeks post-operation), and control group (right column; 2, 4, 6, 8, and 10 weeks post-operation). Black arrowhead: infiltrating inflammatory cell. Red arrowhead: blood capillary

The representative macroscopic findings following the operation are shown in Fig. 4a. The normal patellar tendon (left column) has a flat shape with a width of approximately 1 mm and a length of approximately 5 mm. In both the PRP group (middle column) and the control group (right column), the healing site was covered with a translucent membranous tissue and thickening of the entire tendon tissue was noted 2 weeks post-operation. Thereafter, the defect was gradually obscured and the thickened tendon was gradually remodeled over time up to 10 weeks post-operation. There appeared to be no gross difference in the healing process between the two groups.

The representative histological findings are shown in Fig. 4b-d. The left column shows the normal tendon structure in which the defect has not been created. In the normal tendon specimen, H&E and Azan staining revealed that the collagen fibers were arranged in tightly cohesive, well-demarcated bundles with a smooth, dense, and bright homogeneous polarization pattern. The inconspicuous blood vessels coursing between bundles were present and the tenocytes had inconspicuous elongated spindle-shaped nuclei with no obvious cytoplasm (Fig. 4b, c, left column). Moreover, Alcian blue staining revealed no stainable ground substance (Fig. 4d, left column). In the early phase post-operation, the injured site consisted of loose-granulation tissue with inflammatory cells (black arrowhead in the Azan-stained section). Marked increase in neovascularization (red arrowhead in the Azan-stained section), marked increase in cellularity, marked reduction in collagen fiber alignment, and abundant mucin (intra-fiber blue-stained area in the Alcian blue-stained section) were noted throughout, with inconspicuous collagen staining in both the PRP and the control groups. However, the damaged collagen fibers were aligned and became closer to resembling a normal tendon structure over time in both the PRP and the control groups. The invasion of inflammatory cells, increase of blood capillaries, and thickening of tendon during the early phase seemed more vigorous and collagen re-arrangement was seen earlier in the PRP group than in the control group (Fig. 4b-d, middle and right column).

### Semi-quantitative and quantitative histological analysis

To evaluate the effect of PRP on healing process, we took advantage of the Bonar score (higher grades indicating worse tendon structure) as semi-quantitative evaluation and the ‘FT-ratio’ (the ratio of collagen fibers to the entire tendon tissue, with high percentage indicating better tendon structure) as quantitative evaluation.

**Fig. 5:**
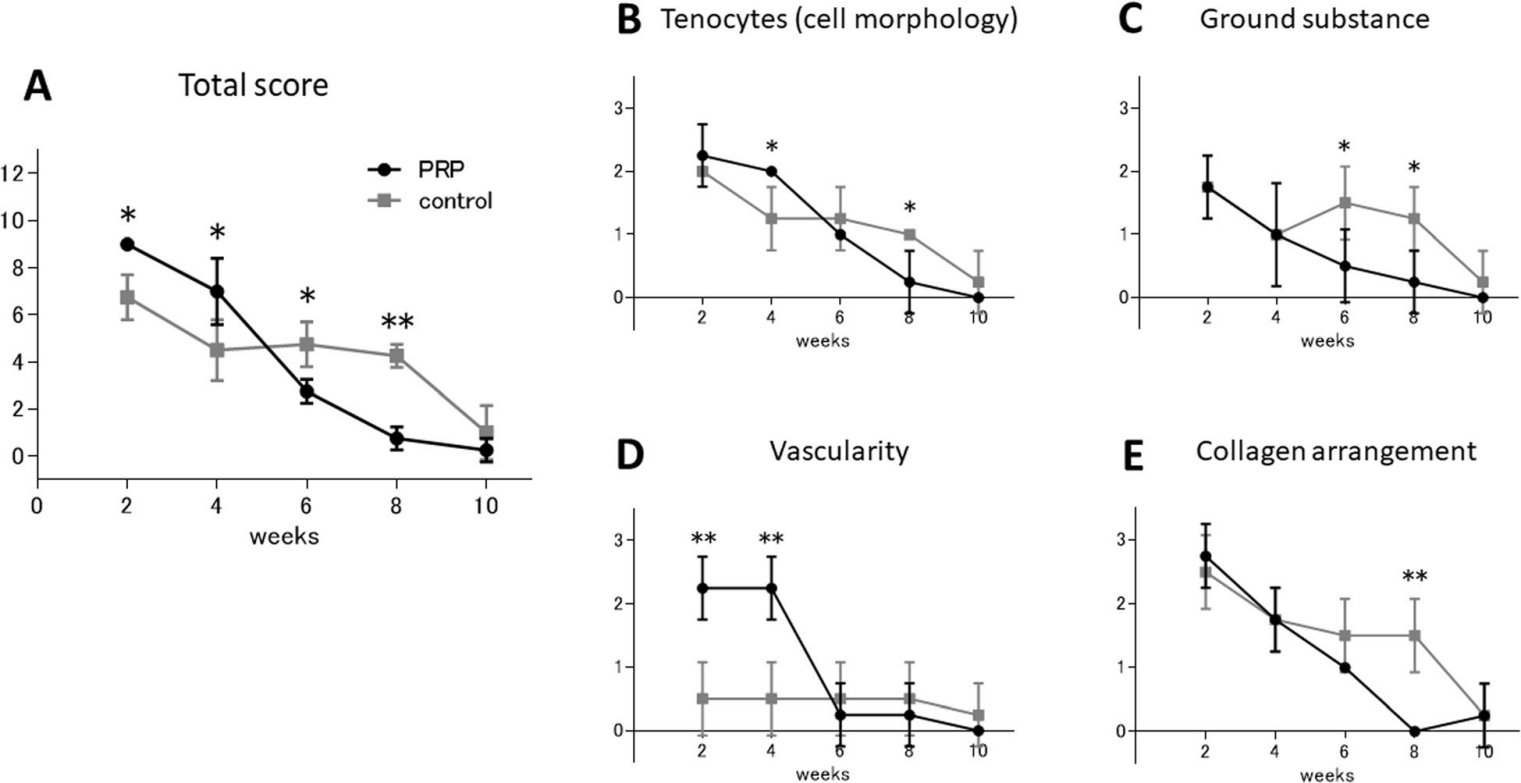
Time-dependent change of Bonar score of PRP and control group. **a** Total score. **b**- **e** Each variable; **b** Tenocytes (cell morphology), **c** Ground substance, **d** vascularity, **e** collagen arrangement. (each factor; 0–3 points, total score; 0–12 points, with higher grades indicating worse tendon structures). Data are presented as mean ± SD (*p < 0.05, **p < 0.01)

In the semi-quantitative evaluation, the total Bonar score and each variable score decreased with time in both the PRP and the control group, indicating get closer to normal tendon structure in both groups with time (Fig. 5a-e). The total Bonar score was significantly higher in the PRP group at 2 and 4 weeks after surgery (*p* = 0.003, *p* = 0.040, respectively), whereas it was significantly lower in the PRP group at 6 and 8 weeks (*p* = 0.010, *p* < 0.001, respectively). There was no significant difference at 10 weeks between the two groups (Fig. 5a). With regard to the variables in the Bonar score, the tenocyte morphology score was significantly higher in the PRP group at 4 weeks (*p* = 0.024), whereas it was significantly lower in the PRP group at 8 weeks (*p* = 0.024) (Fig. 5b). The ground substance score was significantly higher in the control group at 6 and 8 weeks (*p* = 0.050, *p* = 0.030, respectively) (Fig. 5c). The vascularity score was significantly higher in the PRP group at 2 and 4 weeks (*p* = 0.004) (Fig. 5d) and the collagen arrangement score was significantly lower in the PRP group at 8 weeks (*p* = 0.002) (Fig. 5e). These results indicated that administration of PRP significantly increase angiogenesis during early phase of healing process and shorten collagen re-arrangement after the injury.

**Fig. 6:**
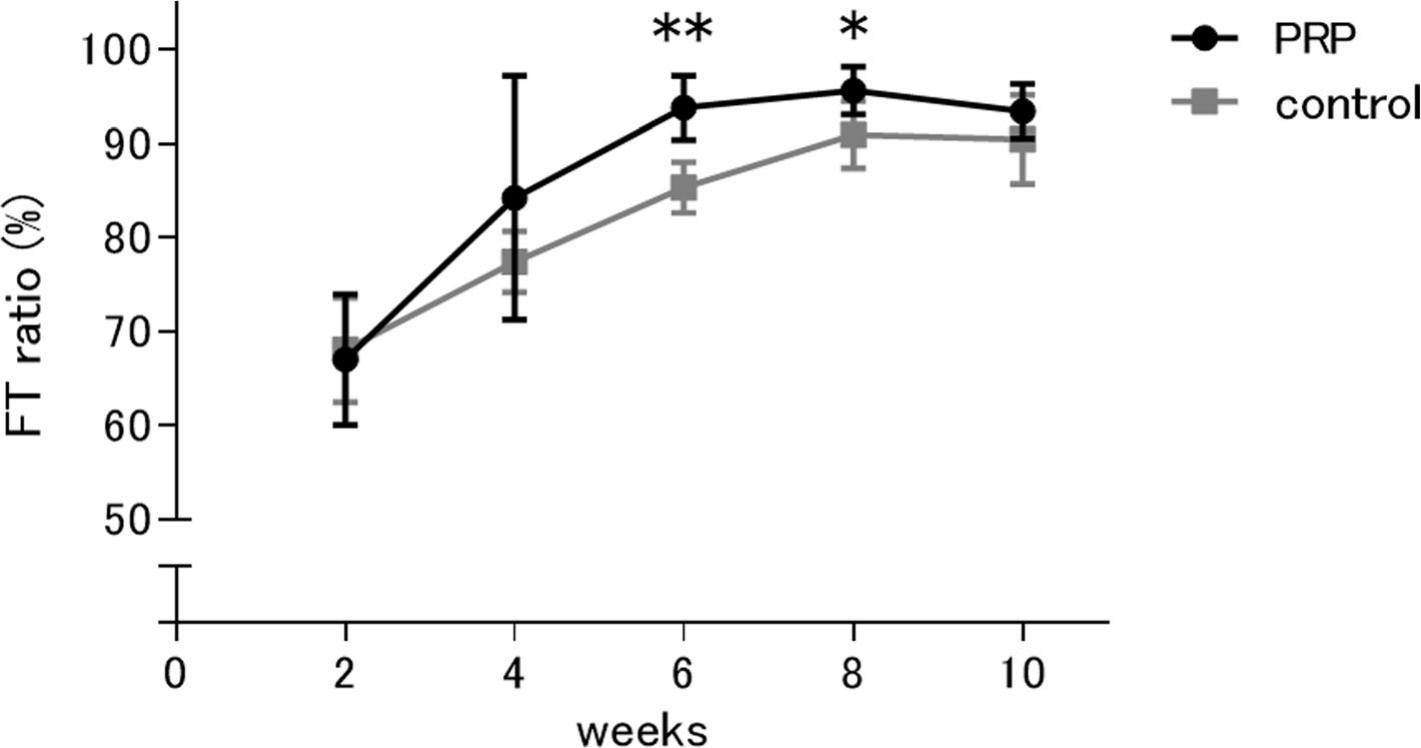
Time-dependent change in the FT ratio of the PRP and control group. FT ratio: the ratio of collagen fibers stained with Azan to the entire tendon tissue. FT ratio was measured using the KS400 image analyzing software (Carl Zeiss, Oberkochen, Germany). The percentages of collagen fiber area positively stained with Azan in the region of interest (ROI) manually drawn along the tendon periphery were determined by computer-assisted morphometry, with a high percentage indicating better tendon structure containing rich collagen fiber as closer to normal tendon. Data are presented as mean ± SD (*p < 0.05, **p < 0.01)

In the quantitative evaluation, the FT ratio increased with time in both groups. The recovery speed of the patella tendon determined by the FT ratio was significantly faster in the PRP group than in the control group at 6 and 8 weeks (*p* = 0.002, *p* = 0.046, respectively) (Fig. 6). This result suggested that administration of PRP significantly fasten the patellar tendon healing.

## Discussion

In this study, we developed an experimental method for the histological and quantitative evaluation of the effects of PRP on tissue healing using a murine patellar tendon injury model. Using this model, we could confirm the efficacy of PRP on murine patellar tendon healing. The semi-quantitative and quantitative histological findings showed that angiogenesis during the early phase of the healing process was significantly increased and collagen re-arrangement after the tendon injury was shortened in the PRP group compared with the control group.

Tendon injuries, both of chronic tendinopathies and acute ruptures, are frequent clinical problems, especially in the field of sports medicine, as they prevent athletes from returning to play. Tendinopathy has been defined as a failure of the healing process [30]. It has been reported that degenerative tendinopathies could lead to tendon rupture [31]. The natural healing process of tendons is slow due to their hypocellular and hypovascular structure [31, 32]. PRP therapy has become an increasingly popular treatment for sports-related injuries, as it could promote tissue healing safely and easily [2]. There are several reports that support the efficacy of PRP therapy for tendon injury [12, 14, 16], but the molecular mechanisms of PRP in the tissue-healing process remain poorly understood. Indeed, one of the major problems is the insufficient evidence supporting the use of PRP in the clinical setting. Abat F et al. posited that basic science studies may be the key to bringing the biological rationale of PRP into safe clinical usage and that preclinical observations may provide well-defined evidence of the mechanism of PRP [33]. To the best of our knowledge, none of the previous in vivo studies regarding the efficacy of PRP have used a murine patellar tendon injury model. There is a possibility of elucidating the mechanisms of PRP in healing processes using this murine patellar tendon injury model because it has the capability of modifying the genome [19]. This technology enables the comparison of tissue properties in mice with and without the ability to express a particular gene globally.

The tissue-healing process is traditionally divided into three sequential and overlapping phases, inflammation, proliferation, and remodeling [30, 31]. Platelets are the first cells that accumulate at the injured site and initiate the healing process by secreting various cytokines and growth factors [34]. In the inflammatory phase, inflammatory cells including neutrophils, monocytes, and lymphocytes migrate from surrounding tissues into the wound site. These cells and platelets produce and secrete the growth factors involved in neovascularization, such as VEGF and PDGF, as well as profibrotic factors, such as TGF-β and bFGF [30]. Following angiogenesis, the intrinsic tenocytes and fibroblasts of surrounding tissues migrate to the injured site and begin proliferating and synthesizing collagen, mainly type III collagen. In the remodeling phase, a higher proportion of type I collagen is synthesized and collagen fibers become aligned, with a decrease in cellularity and type III collagen. PRP has been used based on the hypothesis that the delivery of several molecules will activate the tendon-repair process [30]. However, we should consider the quality of the PRP used. Many of the controversies regarding PRP therapy are based on the absence of a clear definition [35] and the lack of standard PRP preparation procedures or methods of application [33]. There are numerous PRP preparation methods but the differences in PRP quality among them remain unclear. Indeed, our previous work showed that the leukocyte concentration and composition in the PRP influence the expression of growth factors and cytokines [36]. Recently, some classification systems based on the presence or absence of leukocytes, activation status, and platelet concentration have been proposed [10, 35, 37]. Dohan et al. proposed a classification system that divides many products into four main families according to their fibrin architecture and the presence of leukocytes [37]. Given that we prepared the PRP by double-spinning method including the buffy coat to obtain an abundant number of platelets, the PRP used in this study contained leukocyte and erythrocyte, as well as platelet. Thus, this PRP could be categorized as leukocyte rich (LR)-PRP under that classification. There have been some discussions stating that the efficacy of the PRP in tendon healing is affected by the concentration and composition of leukocytes in the PRP, particularly in LR-PRP versus leukocyte poor (LP)-PRP [38]. Some studies have suggested that LR-PRP possesses both catabolic and anabolic effects [39, 40], whereas LP-PRP exerts anabolic effects rather than catabolic effects in injured tissues [41]. It should be noted that the ‘LR-PRP’ used in this study was lymphocyte rich-PRP rather than leukocyte rich-PRP. However, the PRP contained an abundant number of both lymphocyte and granulocyte, with the composition of leukocyte in the PRP being predominantly lymphocyte (88.2%). Unlike humans, lymphocyte is the predominant leukocyte in most strains of healthy wild-type mice [42]. The effect of lymphocytes on tissue healing is also controversial. Linfert D et al. indicated that conventional T-cells are most likely detrimental for tissue healing [43], whereas Li J et al. described that regulatory T-cells (Treg) promote tissue healing and regeneration by modulating inflammation [44]. Although this study could not evaluate a fraction of the lymphocytes, it could have had a positive effect on tissue healing, as the PRP group demonstrated a faster healing process than the control group.

In this study, the Bonar vascularity score was significantly higher in the PRP group at 2 and 4 weeks after the operation than in the control group. This result indicated that the administration of PRP significantly increased angiogenesis during the early phase of the tendon-healing process and that one of the mechanisms of PRP in accelerating tendon-healing processes is the enhancement of blood supply to the injured soft tissue. This would be mediated by angiogenic growth factors, such as VEGF and PDGF, contained in PRP. This observation was similar to the findings of Lyras et al. in a previous study involving a rabbit Achilles tendon injury model reported [16]. They described that PRP temporally increases the angiogenetic phase and subsequently leads to a prompt reduction of this phenomenon, thus accelerating the whole tendon-healing process. The early resumption of blood flow would promote the recruitment of reparative cells from the peripheral blood and bone marrow. In addition, we revealed that collagen re-arrangement after tendon injury was shortened in the PRP group. The Bonar collagen arrangement score was significantly lower in the PRP group at 8 weeks and the FT ratio was significantly higher in the PRP group than in the control group at 6 and 8 weeks after the operation. This was caused by the direct stimulation of local stem cells and extra-cellular matrix genes to accelerate collagen synthesis via the profibrotic growth factors, such as TGF-β and bFGF, contained in the PRP. Based on the result of the ELISA, the PRP used in this study contained an abundant amount of the growth factors involved in neovascularization and fibroblast proliferation. Indeed, the concentrations of these growth factors were almost at the same level as those in other reports using murine LR-PRP [20, 21]. Wasterlain et al. reported the association between cell type and growth factor in the PRP [45]. They described that PDGF and VEGF are derived from both platelets and leukocytes, while TGF-β is primarily derived from the platelets. Therefore, PRP would have boosted the healing cascade by coordinating the growth factors from both the platelets and the leukocytes. These results suggest that the local application of PRP could enhance the tissue-healing process both directly through the action on localized cells and indirectly through the recruitment of reparative cells through the blood flow. This hypothesis supports the report of Kajikawa et al. [46], which revealed through their green fluorescent protein (GFP) chimeric rat patellar tendon injury model that locally injected PRP enhanced the contribution of circulation-derived cells to tendon healing in the early phase of the healing process. Moreover, they speculated that the circulation-derived cells were macrophages. Macrophages are believed to play important roles in the early phase of the tissue-repair process. Macrophages are unique effector cells in innate immunity that play critical roles in tissue repair [47, 48]. In addition, macrophages comprise two phenotypically distinct subtypes, i.e., pro-inflammatory macrophages (M1), which promote the inflammation phase, and anti-inflammatory macrophages (M2), which promote the tissue-regeneration phase. The timely shift from M1 to M2 is thought to be crucial for tissue repair. Arnold L et al. demonstrated that inflammatory monocytes were recruited into the injured site after skeletal muscle injury and switched into anti-inflammatory macrophages to support myogenesis using transgenic mice such as CX3CR1^gfp/+^ and CD11b-DTR [49]. These results could lead to the future elucidation of the mechanisms of PRP in tendon-healing processes.

There are several limitations associated with this study. First, we only performed a histological evaluation. We did not perform functional evaluations, such as biomechanical testing. Lyras et al. used a rabbit patellar tendon injury model and reported that PRP application significantly improved the mechanical properties of the regenerative tendon [50]. Additionally, we did not perform molecular techniques, such as gene and protein assays. It would not be possible to ascertain the mechanisms of PRP in the healing process. However, using this experimental method, further investigations will be expected to lead to the elucidation of its mechanisms. Second, we used allogeneic instead of autologous PRP in this study. Approximately 1 ml of whole blood sampling via cardiac puncture is fatal to mice. Therefore, we could not use the allogeneic PRP in this model. However, these inbred animals are believed to be nearly identical to each other. Third, the murine acute patellar tendon defect model used in this study would not truly replicate the human chronic degenerative condition. Differences exist between animals and humans, but Hast et al. mentioned that animal models allow researchers to understand the cellular and tissue-level principles in the context of a living organism [19].

## Conclusions

We have developed an experimental method for the histological and quantitative evaluation of the effects of PRP on tissue healing using a murine patellar tendon injury model. The results of this study show that angiogenesis during the early phase of the healing process is significantly increased and collagen re-arrangement after tendon injury is shortened in the PRP group compared with the control group. In conclusion, the local application of PRP could enhance tendon healing through the acceleration of vascularization and collagen synthesis. Further investigations will be needed to confirm the mechanisms of PRP in tissue-healing processes with the development of this experimental model.

## Data Availability

The datasets used and analyzed during the current study are available from the corresponding author upon reasonable request.
